# Use of palm bran (*Nopalea cochenillifera* (L.) Salm-Dyck) in partial replacement of concentrate in maintenance equine diets – a pilot study

**DOI:** 10.5194/aab-64-273-2021

**Published:** 2021-06-21

**Authors:** Paula Gomes Rodrigues, Diana Silva Maynard Garcez, Camilla Mendonça Silva, Camilla Cristina Santos Santana, Juliana Caroline Santos Santana, Claudia da Costa Lopes, Evandro Neves Muniz, Gregório Murilo de Oliveira Júnior, Raquel Silva de Moura, José Camisão de Souza

**Affiliations:** 1 Department of Animal Science, Federal University of Sergipe, São Cristovão 49100-000, Brazil; 2 Department of Animal Science, Federal University of Rio Grande of Norte, Macaíba 59.280-000, Brazil; 3 Embrapa Tabuleiros Costeiros, Aracaju 49040-490, Brazil; 4 Department of Animal Science, Federal University of Lavras, Lavras 37-200-000, Brazil

## Abstract

Forage palm is extremely suitable as animal fodder due to its high tolerance to the climatic rigors of the semiarid region and its ability to withstand the harsh physical–chemical limitations of poor soils. Thus, in this study, the effects of the partial replacement (0 %, 5 %, 10 % and 15 % replacement) of a molasses- or oat-based commercial concentrate with forage palm bran (FPB) on the acceptability, apparent digestibility and glycemic response of horses at maintenance were evaluated. The ratio of concentrate to roughage (Tifton 85 hay) was 30:70, and the dry matter (DM) intake was 2 % of body weight (BW). For the preference test, 10 barren Mangalarga Marchador mares were used. The experimental diets were offered simultaneously to determine the consumption preference and the intake ratio. For the digestibility test, four mixed-breed geldings were used and were distributed in a Latin square experimental design (4×4). For the glycemic response, blood samples were collected 30 min before and 30, 60, 90, 120, 180 and 240 min after supplying the feed. The preference test indicated that feed containing 0 % and 5 % FPB was preferred by the animals. Nutrient digestibility coefficients did not differ among the experimental diets. Blood glucose was lower at 180 min in the 7.42 % FPB inclusion diet ($R^{2}=0.97$); this was estimated using the following equation: $Y=115.05-2.75x+0.19x^{2}$. It is concluded that the incorporation of up to 15 % of forage palm bran as a substitute for concentrate in the maintenance diet tested did not negatively influence feed intake, nutrient digestibility or glycemic index; however, inclusion values above 5 % reduced diet acceptability.

## 1 Introduction

Forage palm is extremely suitable as animal fodder due to its high tolerance to the climatic rigors of the semiarid region and its ability to withstand the harsh physical–chemical limitations of poor soils. Forage palm use as feed for maintaining various domestic species (Torres et al., 2009; Sousa et al., 2018), including horses (Velázquez et al., 2016; Parra-Garcia et al., 2018), during prolonged droughts is becoming increasingly more common. In its natural form, this roughage is an excellent source of water for animals; however, in bran form, it may be considered as an energy concentrate due to the low concentration of its cell-wall constituents (Peixoto et al., 2018). The most widespread forage palm species is the small sweet palm (*Nopalea cochenillifera* (L.) Salm-Dyck), which is easy to plant and harvest and has greater acceptability (Carvalho et al., 2018) – a characteristic attributed to its nutritional composition.

Recent studies have highlighted that the *Miúda* cultivar of forage palm (*Nopalea cochenillifera* (L.) Salm-Dyck) has an average of 11.9 % dry matter (DM) as well as (based on DM) 4.4 % crude protein (CP), 27.3 % neutral detergent fiber (NDF), 17.3 % acid detergent fiber (ADF) and 1.8 % ether extract (EE) (Silva et al., 2017; Carvalho et al., 2018). Palm bran also has a low starch concentration and a high content of soluble carbohydrate; pectin; vitamins A, B, E and C; and minerals such as calcium, magnesium and potassium (Nunes et al., 2011; Neto et al., 2016).

These characteristics indicate the great potential of using palm bran in the diet of horses that, as functional cecum herbivores, are able to efficiently utilize its carbohydrates through cecal microbial fermentation (Elghandour et al., 2018). In addition, horses are susceptible to several metabolic disorders caused by excess starch in their diet; therefore, the use of feeds containing low starch becomes essential for maintaining the health of the digestive tract (NRC, 2007). Excess starch also promotes elevated postprandial glycemic responses, which can cause diseases such as insulin sensitivity (Julliand et al., 2018). Another advantage of the inclusion of palm bran in the diet is its lower cost compared with corn bran (Pascoal et al., 2020).

There are few studies reporting the use of palm bran (Parra-Garcia et al., 2018) in horse diets, especially with respect to its acceptability and digestibility. Horses are highly selective animals: they select foods based on factors such as visual characteristics, odor, flavor, availability, texture and variety (Janczarek et al., 2018). Thus, when evaluating the use of an alternative feed, variables such as intake, digestibility and carbohydrate metabolism should be optimized, as they impact performance directly. The following two hypotheses were made in this study: (1) the inclusion of palm bran in the concentrate of equine diets does not decrease intake, and (2) the digestibility coefficient and the postprandial glycemic index are similar between diets containing different levels of palm bran inclusion and control diets (without palm bran). Thus, the objective this pilot study was to evaluate the effects of the partial replacement of a commercial concentrate with forage palm bran on acceptability, apparent digestibility and glycemic response in maintenance equine diets.

## 2 Material and methods

All experimental procedures were approved by the Ethics Committee in Research with Production Animals of the Federal University of Sergipe according to protocol no. 05/15.

### 2.1 Forage palm bran (FPB) production

The small sweet (*Nopalea cochenillifera* (L.) Salm-Dyck) forage palm cultivar was used. The palm was obtained from the Embrapa Coastal Tablelands experimental station, located in Frei Paulo, Sergipe, Brazil (10${}^{\circ }$35${}^{\prime }$01.2${}^{\prime \prime }$ S, 37${}^{\circ }$37${}^{\prime }$18.6${}^{\prime \prime }$ W; 272 m altitude). Cladodes that were up to 1 year old and showed no signs of rot or withering were used. The cladodes were chopped into thin slices (about 3 cm thick) and were spread on greenhouse trays in an even layer. The drying process was carried out in an oven with forced ventilation at 65 ${}^{\circ }$C for 72 h. After removal from the oven, the material was ground in a knife mill with a 1 mm sieve and then stored in a duly identified plastic bag and kept in a moisture-free environment.

### 2.2 Experimental diets

The experimental diets were formulated to meet the nutritional requirements of adult horses at maintenance according to the NRC (2007) recommendations. Free access to Tifton 85 hay (*Cynodon* spp.) and a commercial pelleted concentrate, in the quantities recommended for adult horses at maintenance (NutriEqui PMSE^®^15.6), were offered daily, and water and mineral mix were offered ad libitum (Guabiphos Centauro 80^®^, Guabi Animal Nutrition) (Tables 1, 2).

**Table 1:** Chemical composition of diet components (based on dry matter, DM)

Nutrient	Components		
	Concentrate${}^{1}$	FPB	Tifton 85 hay (*Cynodon* spp.)
Dry matter (%)	87.07	93.14	83.80
Organic matter (%)	91.97	74.60	93.76
Mineral (%)	8.03	25.40	6.24
Ether extract (%)	6.07	0.98	0.76
Crude protein (%)	11.08	2.62	6.16
NDF (%)${}^{2}$	39.64	51.69	84.07
ADF (%)${}^{3}$	15.36	23.70	54.83
Gross energy (Mcal/kg)	4.35	2.87	4.21
Digestible energy (Mcal/kg)${}^{4}$	3.225	2.766	2.001
Hemicellulose (%)	24.27	27.98	29.24
Nonstructural carbohydrate (%)${}^{5}$	35.17	19.32	2.76

${}^{1}$ NutriEqui PMSE 15.6, Guabi^®^ Nutrição Animal. ${}^{2}$ NDF denotes neutral detergent fiber. ${}^{3}$ ADF denotes acid detergent fiber. ${}^{4}$ Digestible energy (Mcal/kg) was calculated using the equation proposed by NRC (2007): DE${}_{\mathrm{concentrate}}$ (Mcal/kg) = 4.07 – 0.055 × (ADF %), and DE${}_{\mathrm{roughage}}$ (Mcal/kg) = 4.22-0.11 × (ADF %) + 0.0332 × (CP %) + 0.0012 × (ADF %${}^{2})$. ${}^{5}$ Nonstructural carbohydrate (%) was calculated using the equation proposed by Hoffman (2001).

**Table 2:** Chemical composition of the total diets composed of commercial concentrate${}^{1}$, forage palm bran and Tifton 85 hay (*Cynodon* spp.).

Nutrient	FPB inclusion in diets (in %)			
	0	5	10	15
Dry matter (%)	84.78	84.87	84.96	85.06
Organic matter (%)	93.22	92.96	92.70	92.44
Mineral matter (%)	6.78	7.04	7.30	7.56
Ether extract (%)	2.36	2.28	2.20	2.13
Crude protein (%)	7.64	7.51	7.38	7.25
NDF (%)${}^{2}$	70.74	70.92	71.10	71.28
ADF (%)${}^{3}$	42.99	43.12	43.24	43.37
Gross energy (Mcal/kg)	4.25	4.23	4.20	4.18
Digestible energy (Mcal/kg)${}^{4}$	2.368	2.361	2.354	2.347
Hemicellulose (%)	27.75	27.80	27.86	27.91
Nonstructural carbohydrate (%)${}^{5}$	12.49	12.25	12.01	11.77

${}^{1}$ NutriEqui PMSE 15.6, Guabi^®^ Nutrição Animal. ${}^{2}$ NDF denotes neutral detergent fiber. ${}^{3}$ ADF denotes acid detergent fiber. ${}^{4}$ Digestible energy (Mcal/kg) was calculated using the equation proposed by NRC (2007): = 4.07 - 0.055 × (ADF %), calculated values (NRC, 2007). ${}^{5}$ Nonstructural carbohydrate (%) was calculated using the equation proposed by Hoffman (2001).

For the preference test and digestibility test, four treatments were applied:

- *Treatment 1* (control) – no FPB;
- *Treatment 2* – inclusion of 5 % (50 g/kg) FPB as a substitute for commercial concentrate;
- *Treatment 3* – inclusion of 10 % (100 g/kg) FPB as a substitute for commercial concentrate;
- *Treatment 4* – inclusion of 15 % (150 g/kg) FPB as a substitute for commercial concentrate.

### 2.3 Preference test

For the intake preference test, 10 barren, healthy, 10 ± 4-year-old Mangalarga Marchador mares weighing 355 ± 25 kg were used. Mares were kept using an extensive all-pasture system (Bermuda grass – *Cynodon dactylon* (L.) Pers.). Animals scored from 4 (moderately thin) to 6 (moderately conditioned) according to a body condition scale (BCS) developed by Henneke et al. (1983), which ranges from 1 (extremely thin) to 9 (extremely obese). Mares were allocated to treatments in a completely randomized pattern.

The preference test was divided into two steps: in Step I, the commercial concentrate was offered in pelleted form; in Step II the concentrate was offered as bran after processing in a 3 mm sieve mill. The four treatments were the same in both steps. The step interstitial period was 30 d.

The diets were provided in a single concrete trough that was 3 m in length. The treatments (individual feed portions) were arranged so as to be equidistant from each other along the trough and in a random manner, minimizing the possibility of an animal choosing any specific region (Tribucci et al., 2013).

The preference test was performed according to the methodology proposed by Rivera et al. (2019). For both stages, the experimental period lasted 6 d: 3 d for adaptation and 3 d for observations. All animals were evaluated on each of the 3 observation days, resulting in 30 observations for each stage. The instantaneous focal methodology was used (Martin and Bateson, 1986), and each animal was observed for 15 min. The order of the animals' choice of treatments was recorded – the treatment that was effectively ingested, not just smelled by the animal, was considered to be the one that was chosen or preferred.

The animals were removed from the paddock and taken into the individual observation stalls. Before the test, all mares received 300 g of pelleted (Step I) or bran (Step II) commercial feed without palm forage in order to reduce anxiety (Dittrich et al., 2010). There was then a 10 min interval between the end of this meal and the start of the preference test.

During the preference test, 300 g of feed from each treatment was provided. Each experimental diet was randomly placed in the trough (so as to be equidistant from the other treatments), and the treatments were offered simultaneously to the animals, resulting in a total feed mass of 1.2 kg.

At the end of each observation, in Step I, orts were weighed in order to determine the intake ratio (IR). The IR percentage was obtained by the ratio between the amount actually consumed in a diet in relation to the total amount of feed offered, considering the four treatments (Rivera et al., 2019); this was calculated as follows:

1 $$\mathrm{IR}=\frac{\text{intake}\left(g\right)\text{diet A}}{\left(g\right)\text{diet A+B+C+D}}.$$

Intake ratios >0.25 were assigned as the limit to determine the most accepted diet. Preference was calculated by the sum of the orders of choice for each diet, which received values from one to four, where the first treatment chosen received a value of one and so on; therefore, the concentrate chosen first received the lowest score.

### 2.4 Apparent digestibility assay

For the digestibility test, four healthy 13 ± 2-year-old adult Mangalarga Marchador horses with a weight of 449 ± 15 kg, a withers hight of 152 ± 5 cm, and a BCS from 4 to 6 were chosen. All treatments were applied over time in a Latin square design (4×4).

Animals were kept in individual stalls (16 m${}^{2}$ per animal). The stalls were equipped with a water trough and three feed troughs to allow for concentrate, roughage and mineral mix feed, respectively. The ratio of concentrate to roughage was 30:70, and the dry matter intake was 2 % of the mean body weight for adult horses at maintenance (NRC, 2007). The concentrate was offered twice a day (04:00 and 14:30 LT, local time), and roughage was offered in three daily portions of different amounts: 30 % at 07:00 LT, 30 % at 11:00 LT and 40 % at 17:00 LT. The amount (kg) of feed offered daily under normal maintenance conditions was 10 kg. The control animals, without FPB, received 3.0 kg of the commercial concentrate and 7 kg of roughage. The animals receiving the palm bran treatment were fed 2.85 kg of a commercial pelleted concentrate, 0.150 kg of FPB and 7 kg of roughage. In the 10 % FPB inclusion treatment, animals were fed 2.70 kg of the pelleted commercial concentrate, 0.30 g of FPB and 7 kg of roughage. The 15 % FPB group received 2.55 kg of the commercial pelleted concentrate, 0.45 kg of FPB and 7 kg of roughage.

The digestibility test lasted 10 d: 7 d were used for adaptation, and 3 d were used for the total feces collection. During the experimental period, the concentrate and roughage offered as well as the orts were quantified to calculate the feed intake and digestibility coefficients.

Feces were collected immediately after excretion over the bedding; at the end of each day they were weighed and homogenized, and a 15 % aliquot (subsample) was stored and frozen at -10 ${}^{\circ }$C. At the end of the experimental period, the subsamples were thawed and homogenized to compose a single sample for each treatment, for the determination of bromatological analyses.

The apparent digestibility coefficient (DC${}_{\mathrm{ap}}$) was estimated according to Andriguetto and Perly (1981); it was calculated as follows:

2 $${\mathrm{DC}}_{\mathrm{ap}}\;\%=\left[\frac{{\mathrm{Nutrient}}_{\mathrm{ingested}}-{\mathrm{Nutrient}}_{\mathrm{feces}}\cdot 100}{{\mathrm{Nutrient}}_{\mathrm{ingested}}}\right].$$

Nutrient dry matter digestibility coefficients (DMDC${}_{\mathrm{ap}}$), organic matter (OMDC${}_{\mathrm{ap}}$), ether extract (EEDC${}_{\mathrm{ap}}$), crude protein (CPDC${}_{\mathrm{ap}}$), neutral detergent fiber (DNFDC${}_{\mathrm{ap}}$), acid detergent fiber (ADFDC${}_{\mathrm{ap}}$), hemicellulose (HEMDC${}_{\mathrm{ap}}$) and nonstructural carbohydrates (NSCDC${}_{\mathrm{ap}}$) were determined according to Inácio et al. (2017).

Apparent digestible energy (DE${}_{\mathrm{ap}}$) was determined according to Oliveira et al. (2002):

3 $${\mathrm{DE}}_{\mathrm{ap}}\left(\mathrm{Kcal}/\mathrm{kg}\right)=\frac{\left[\mathrm{GEI}-\mathrm{FCE}\right]}{\mathrm{DMI}},$$

where DMI is daily dry matter intake, GEI is gross energy intake and FCE is fecal crude energy.

### 2.5 Bromatological analyses

Nutrient dry matter (DM-Method 934.01; AOAC Int., 2012), mineral matter (MM-Method 984.08; AOAC Int., 2012), ether extract (EE-Method 920.39; AOAC Int., 2012), crude protein (CP-Method 992.15; AOAC Int., 2012), mineral matter (MM) and ether extract (EE) were determined using the methodologies described in AOAC (2012). An adiabatic calorimeter (C-200, IKA^®^ Works) was used the determine the gross energy (GE), with benzoic acid as the standard calibrator. The analyses of neutral detergent fiber (NDF) and acid detergent fiber (ADF) were performed as described by Van Soest et al. (1991).

Fractions of nonstructural carbohydrate (NSC) were determined according to Hoffman (2001) (Table 1).

The digestible energy values were estimated according to the formulas described in the NRC (2007) for concentrate and roughage:

4DEconcentrateMcal/kg=4.07-0.055⋅(ADF%),5DEroughage(Mcal/kg)=4.22-0.11⋅ADF%+0.0332⋅CP%+0.0012⋅(ADF%2).

### 2.6 Glycemic response

Blood glucose samples were taken during the digestibility test, on the last day of the total feces collection. The collection periods were 30 min before and 30, 60, 90, 120, 180 and 240 min after the experimental feeds were given, according to the methodology described by Rodiek and Stull (2007).

After local antisepsis, blood samples were obtained through venipuncture of the jugular using disposable needles, in 2 mL vacuum collection tubes containing anticoagulant ethylenediaminetetraacetic acid (EDTA)–sodium fluoride, for plasma glucose assessment; 4 mL of blood was collected and centrifuged, and 2 mL of plasma was stored in microtubes and frozen at -10 ${}^{\circ }$C. Plasma glucose concentrations were determined in a biochemical analyzer (Thermo plate, TP Analyzer Basic) using a Glucose Liquiform (Glucose Liquiform, Labtest Diagnóstico SA, Lagoa Santa/MG, Brazil) reagent via the enzymatic colorimetric method.

For the glycemic test, mean plasma glucose concentrations, peak responses and the time to reach the glucose peaks were calculated. Mean glucose concentrations were analyzed using the area under the curve (AUC), considering the baseline and postprandial moments (0 to 240 min) according to Borghi et al. (2017).

### 2.7 Statistical analyses

All data were analyzed using the Sisvar system (Ferreira, 2019). Intake preference tests were evaluated using the Friedman test, with data from the table of Newell and Macfarlane (Meilgaard et al., 1991). Intake ratio (IR) data were subjected to an analysis of variance and regression analysis. For intake, apparent digestibility and glycemic responses, the fixed effects of treatments (0 %, 5 %, 10 % and 15 % of FPB), period and random animal within the Latin square and residual were considered. The first value for each variable observed within the experimental period was used as a covariate. Variance and regression analyses used the 5 % level of significance, and means were compared using a Tukey test.

## 3 Results

### 3.1 Preference test

**Figure 1:**
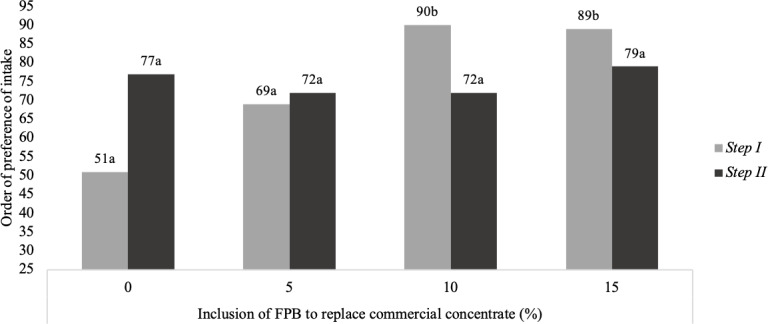
Preference test and intake of diets with 0 %, 5 %, 10 % and 15 % of forage palm bran (FPB) replacement of commercial concentrate in Step I (pelleted concentrate) and Step II (ground concentrate). Different superscripts denote a significant difference (P<0.05), according to the Newell and Macfarlane table (Meilgaard et al., 1991). The standard error of the mean was 9.28 in Step I and 1.78 in Step II.

In Step I (pelleted concentrate), the preference test indicated that the diets with low FPB inclusion (0 % and 5 %) were preferred (P<0.01) compared with the other treatments (Fig. 1). However, when FPB was added to the concentrate in Step II (commercial concentrate), there was no difference with respect to preference.

**Figure 2:**
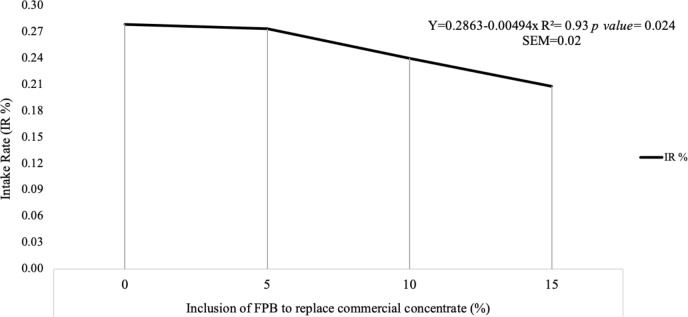
Intake ratio (IR %) of diets with four levels of inclusion of forage palm bran (FPB) to replace a commercial concentrate. SEM denotes the standard error of the mean.

**Figure 3:**
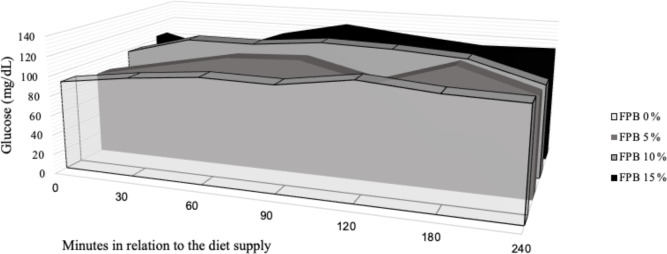
Blood glucose concentrations (area under the curve) considering the baseline and post-feeding (0–240 min after feeding) levels in horses fed four inclusion levels of palm bran in maintenance diets.

Corroborating these results, the intake ratio (Fig. 2) was associated with a lower preference for the 10 % (IR = 0.2399) and 15 % (IR = 0.2080) inclusion diets.

### 3.2 Diet intake

The inclusion of different levels of FPB did not affect total DM intake and DM intake in relation to the BW (P>0.05; Table 3). The intakes of organic matter (OM), CP, NDF, ADF, gross energy (GE), digestible energy (DE), hemicellulose and NSC were not different (P>0.05) among treatments. However, there was a linear increase (P=0.005) in MM intake and a reduction (P=0.006) in EE intake with increases in the levels of FPB inclusion (Table 4).

**Table 3:** Final mean body weight (BW); dry matter intake (DMI) with respect to concentrate, roughage, forage palm bran and total intake; and daily total dry matter intake relative to body weight (TDMI).

Variables	FPB inclusion in diets (in %)				$P^{1}$	CV${}^{2}$
	0	5	10	15		
Body weight (kg)	459	458	458	463	0.70	1.64
DMI${}_{\mathrm{concentrate}}$ (kg)	2.75	2.60	2.38	2.35	0.96	2.41
DMI${}_{\mathrm{roughage}}$ (kg)	5.94	5.75	5.89	5.87	0.83	2.63
DMI${}_{\mathrm{FPB}}$ (kg)	0.00	0.15	0.29	0.44	0.74	1.26
DMI${}_{\mathrm{total}}$ (kg)	8.69	8.50	8.56	8.66	0.90	4.74
TDMI (% BW)	1.89	1.86	1.87	1.87	0.95	5.59

${}^{1}$ P value calculated using a Tukey test. ${}^{2}$ Coefficient of variation.

**Table 4:** Total daily intake of nutrients from diets enriched with different levels of forage palm bran inclusion (FPB %).

Intake	FPB inclusion in diets (in %)				$P^{1}$	CV${}^{2}$
	0	5	10	15		
OMI${}_{\left(\mathrm{kg}\right)}$	8.15	7.94	7.97	8.03	0.8755	4.86
MMI${}_{\left(\mathrm{kg}\right)}$	0.59	0.60	0.63	0.67	0.005${}^{a}$	4.22
EEI${}_{\left(\mathrm{kg}\right)}$	0.22	0.21	0.19	0.19	0.006${}^{b}$	3.92
CPI${}_{\left(\mathrm{kg}\right)}$	0.68	0.65	0.64	0.64	0.3204	4.57
NDFI${}_{\left(\mathrm{kg}\right)}$	6.11	5.96	6.06	6.11	0.9028	5.25
ADFI${}_{\left(\mathrm{kg}\right)}$	3.69	3.60	3.67	3.69	0.8974	5.48
GEI${}_{\left(\mathrm{Mcal}/\mathrm{kg}\right)}$	3.7	3.6	3.6	3.6	0.8299	4.78
DEI${}_{\left(\mathrm{Mcal}/\mathrm{kg}\right)}$	2.07	2.03	2.02	2.05	0.8505	4.40
HEMI${}_{\left(\mathrm{kg}\right)}$	2.42	2.37	2.39	2.42	0.9027	4.92
NSCI${}_{\left(\mathrm{kg}\right)}$	1.15	1.12	1.07	1.09	0.4547	0.09

${}^{1}$ P values differ according to a Tukey test (P<0.05). ${}^{2}$ Coefficient of variation. ${}^{a}$ y=0.585733+0.005106x, $R^{2}=0.96$. ${}^{b}$ y=0.213518-0.001482x, $R^{2}=0.91$. The abbreviations used in the table are as follows: OMI – organic matter, MMI – mineral matter, EEI – ether extract, CPI – crude protein, NDFI – neutral detergent fiber, ADFI – acid detergent fiber, GEI – gross energy, DEI – digestible energy intake, HEMI – hemicellulose and NSCI – nonstructural carbohydrates.

### 3.3 Apparent digestibility coefficients

The inclusion of FPB did not change (P>0.05) the apparent digestibility coefficients (Table 5).

**Table 5:** Apparent coefficients of digestibility of nutrients from diets enriched with different levels of forage palm bran inclusion (FPB %).

Coefficients (%)	FPB inclusion in diets (in %)				$P^{1}$	CV${}^{2}$
	0	5	10	15		
DMCD${}_{\mathrm{ap}}$	65.13	59.04	61.40	61.31	0.3533	7.13
OMCD${}_{\mathrm{ap}}$	66.45	60.32	62.85	62.75	0.3471	6.91
EECD${}_{\mathrm{ap}}$	68.55	60.41	60.43	64.17	0.2253	8.77
CPCD${}_{\mathrm{ap}}$	85.15	82.51	79.79	72.91	0.6085	4.86
NDFCD${}_{\mathrm{ap}}$	62.73	55.40	59.04	59.04	0.3703	9.05
ADFCD${}_{\mathrm{ap}}$	54.35	51.06	53.88	54.16	0.7953	9.88
HEMCD${}_{\mathrm{ap}}$	75.16	62.01	66.95	66.40	0.0663	7.99
NSCCD${}_{\mathrm{ap}}$	98.55	98.50	98.62	98.74	0.4744	0.22

${}^{1}$ P values differ according to a Tukey test. ${}^{2}$ Coefficient of variation. The abbreviations used in the table are as follows: DMCD${}_{\mathrm{ap}}$ – apparent digestibility coefficients of dry matter, OMCD${}_{\mathrm{ap}}$ – apparent digestibility coefficients of organic matter, EECD${}_{\mathrm{ap}}$ – apparent digestibility coefficients of ether extract, CPCD${}_{\mathrm{ap}}$ – apparent digestibility coefficients of crude protein, NDFCD${}_{\mathrm{ap}}$ – apparent digestibility coefficients of neutral detergent fiber, ADFCD${}_{\mathrm{ap}}$ – apparent digestibility coefficients of acid detergent fiber, HEMCD${}_{\mathrm{ap}}$ – apparent digestibility coefficients of hemicellulose and NSCCD${}_{\mathrm{ap}}$ – apparent digestibility coefficients of nonstructural carbohydrates.

### 3.4 Postprandial glycemic response

The glycemic response 30 min after feeding increased equally in all treatments (Table 6). Blood glucose remained high until 180 min after feeding, when the peak plasma glucose concentration occurred, converging to baseline values at 240 min in all treatments. The lowest value of the glucose peak was observed 180 min after feeding in the treatment containing 7.42 % FPB inclusion (P=0.04), according to the following equation: $Y=115.05-2.75x+0.19x^{2}$, $R^{2}=0.97$. Baseline plasma glucose, peak glucose concentrations, time (minutes) to plasma glucose peak and AUC (Fig. 3) were similar between treatments (P>0.05; Table 7).

**Table 6:** Pre- and post-feeding blood glucose (mg/dL).

Time (min)	FPB inclusion in diets (in %)				$P^{1}$	CV${}^{2}$
	0	5	10	15		
-30${}^{a}$	98.48	87.96	101.16	94.35	0.3165	9.96
30	111.65	106.03	103.07	100.50	0.6521	11.99
60	119.66	111.35	102.15	111.97	0.2305	9.34
90	125.74	121.15	106.75	117.88	0.3039	11.15
120	120.08	116.63	119.20	113.74	0.9082	11.54
180${}^{b}$	115.41	104.85	107.14	115.13	0.004${}^{**}$	3.75
240	105.21	107.54	105.72	104.49	0.9907	13.31

${}^{1}$ P values differ according to a Tukey test (P<0.05). ${}^{2}$ Coefficient of variation. ${}^{a}$ A total of 30 pre-feeding minutes. ${}^{b}$ $Y=115.05-2.75x+0.19x^{2}$, $R^{2}=0.97$.

**Table 7:** Blood glucose parameters in horses fed four inclusion levels of forage palm bran (FPB) to replace commercial concentrate.

Parameter	FPB inclusion in diets (in %)				$P^{1}$	CV${}^{2}$
	0	5	10	15		
Basal (mg/dL)	98.5	88.0	101.2	94.4	0.3165	9.96
Peak (mg/dL)	130.8	126.3	120.0	121.9	0.1458	9.15
Time to reach peak (min)	75.0	165.0	97.5	120.0	0.3986	62.32
AUC${}^{3}$ (mg/dL × min)	27 673.2	26 240.7	25 840.7	26 486.7	0.1896	4.01

${}^{1}$ P value calculated using a Tukey test. ${}^{2}$ Coefficient of variation. ${}^{3}$ Area under the curve.

## 4 Discussion

It was found, from the preference test, that the increased inclusion of FPB reduced the preference for feeds – that is, the higher the content of palm bran mixed with the concentrated food, the lower its acceptance. However, feed containing palm was not rejected by the animals, having been completely consumed at the end of the observation periods, but it was the last to be chosen (Figs. 1, 2).

This preference for diets containing less forage palm was only found in Step I, when the concentrate was offered in pelleted form. In Step II, when the concentrate was offered in bran form, no difference in preference was observed.

Some studies show that horses have developed a feed intake strategy, selecting their diet based on visual characteristics, odor, flavor, texture, availability and variety, always giving preference to what is familiar (Goodwin et al., 2005; Janczarek et al., 2018).

It is possible that the horses, who were used to ingesting the commercial pelleted feed, reacted negatively to the addition of the palm bran because it modified the structure of the pellet, decreasing its firmness over time. It is also possible that the high pectin content in palm bran may have altered the texture of the commercial diet feed due to its high agglutination capacity (Carvalho et al., 2018), negatively influencing the preference for diets containing higher levels of FPB.

The overall mean DM intake was 1.8 % of the body weight, which is within the NRC (2007) recommendations of 1.5 %–3.0 % for the maintenance of adult horses.

According to Frape (2008), the main factors that regulate DM intake in horses are the capacity of the different segments of the gastrointestinal tract, the rate of digestion passage, the concentration of nutrients in the diet, and, especially, the fulfillment of energy and protein requirements. It is noteworthy that the overall DE and CP intakes met the NRC (2007) requirements, suggesting that this may have been a factor responsible for keeping the DM intake as expected (Table 3).

The higher intake of MM followed the increase in the level of inclusion of FPB, a characteristic related to the high mineral content of this feed (25.40 %) compared with the concentrate (8.03 %). The inverse was observed for the EE intake, which decreased as FPB inclusion increased, due to the low concentration of this nutrient in the bran (0.98 %) compared with that of the concentrate (6.07 %).

As apparent digestibility coefficients were similar among treatments, it is possible to infer that the inclusion of FPB provides an excellent source of nutrients in the diet. This is possibly due to the chemical composition of FPB, which has high concentrations of NDF (51.69 %) and hemicellulose (27.28 %). These nutrients are converted to volatile fatty acids during cecal microbial fermentation providing energy that is rapidly available to the animal (Merrit and Julliand, 2013).

Therefore, even with a reduction in the amount of energy in the diet, as the level of inclusion of FPB increased, there was no decrease in the apparent digestibility coefficients, probably due to the large presence of fermentable carbohydrates in the FPB which compensated for its low density.

Digestibility coefficients lower than those observed were found by Casalecchi et al. (2012) when evaluating a concentrate of extruded corn for adult mares: 44.7 % for NDFDC${}_{\mathrm{ap}}$ and 47.8 % for ADFDC${}_{\mathrm{ap}}$. When studying the digestibility of palm oil for horses, Gobesso et al. (2009) obtained a NDFDC${}_{\mathrm{ap}}$ of 56.84 % and ADFDC${}_{\mathrm{ap}}$ of 51.65 %. Therefore, it can be inferred that FPB has a cell wall that is highly digestible, allowing for the optimization of fermentative activity in the cecum colon and contributing to the fulfillment of the energy requirement of horses by allowing the absorption of volatile fatty acids.

Baseline glucose concentrations were consistent with the physiological concentrations reported in the literature for fasting horses, between 80 and 100 mg/dL, which reached 150 mg/dL at 3 h following the consumption of a starchy diet (Jacob et al., 2018). However, peak glucose concentrations did not reach this value. The peak glucose value, considering treatments containing FPB, was 122.7 mg/dL.

When evaluating diets composed of grass hay and concentrate containing different starch sources (corn, oats and sorghum) in the equine diet, Gobesso et al. (2009) found peak glucose concentrations of 161.63 mg/dL for corn and 123.23 mg/dL for oats 90 min after ingestion of the experimental diets, which are values higher than those observed in the present study (Table 6).

The occurrence of a relatively low value at the time of peak plasma glucose concentration may indicate that the absorption of this nutrient occurred uniformly over time, which is beneficial to the animal in view of its high predisposition to become insulin resistant when fed high starch diets (Olley et al., 2019).

The area under the curve (AUC) indicated that the inclusion of FPB did not increase blood glucose concentration over time. The AUC assists in the quantification of the glycemic response to a certain feedstuff with a known carbohydrate concentration: the sooner the glucose is removed from the bloodstream, the lower the AUC will be (Jacob et al., 2018).

Postprandial glycemic responses are regulated according to several factors, such as chyme viscosity, passage rate prolongation, delayed α-amylase activity and production of volatile fatty acids in the large intestine (Casalecchi et al., 2012). The substitution of part of the starch present in the commercial concentrate for the soluble fiber (pectin) or insoluble fiber (cellulose and hemicellulose) present in palm bran may have been responsible for reducing blood glucose over time, as well as the glucose peak.

The current results demonstrate the potential of FPB as a low-glycemic-index feed for horses, which are usually susceptible to numerous metabolic disorders, such as insulin resistance, obesity, colic and laminitis, resulting from the excessive supply of hydrolyzable carbohydrates in their diet (Olley et al., 2019). The use of feeds with a low glycemic index is a strategy to reduce the occurrence of these serious nutritional disorders (Rodiek and Stull, 2007).

## 5 Conclusions

The horses had a positive acceptance to forage palm bran (*Nopalea cochenillifera* (L.) Salm-Dyck), which could replace up to 15 % of commercial concentrates for horses without reducing their feed intake or decreasing the digestibility of dietary nutrients, keeping the postprandial glycemic response low. Thus, palm bran proved to be an alternative feedstuff of great potential for equine maintenance diets.

## Data Availability

The original data from the paper are available upon reasonable request from the corresponding author.
